# MUC1 in lung adenocarcinoma: cross-sectional genetic and serological study

**DOI:** 10.1186/s12885-017-3272-y

**Published:** 2017-04-12

**Authors:** Yasushi Horimasu, Nobuhisa Ishikawa, Sonosuke Tanaka, Chihiro Hirano, Hiroshi Iwamoto, Shinichiro Ohshimo, Kazunori Fujitaka, Hironobu Hamada, Noboru Hattori, Nobuoki Kohno

**Affiliations:** 1 Department of Molecular and Internal Medicine, 1-2-3 Kasumi, Minami-ku, Hiroshima 734-8551 Japan; 2 Physical Analysis and Therapeutic Sciences, Institute of Biomedical and Health Sciences, Hiroshima University, 1-2-3 Kasumi, Minami-ku, Hiroshima 734-8551 Japan; 3 Department of Respiratory Medicine, Hiroshima Prefectural Hospital, 1-5-54 Ujina-Kanda, Minami-ku, Hiroshima 734-8530 Japan; 4 Department of Internal Medicine, Shobara City Saijo Citizens Hospital, 1339 Nakano, Saijo-cho, Shobara, 729-5742 Japan

**Keywords:** KL-6, MUC1, rs4072037, Lung adenocarcinoma

## Abstract

**Background:**

Mucin 1 (MUC1) contributes to the growth and metastasis of various cancers, including lung cancer, and *MUC1* gene length polymorphisms are associated with susceptibility to lung cancer and its prognosis. In contrast, the association between rs4072037, a single nucleotide polymorphism in *MUC1*, and lung cancer has not been well studied.

**Methods:**

In the present study, we determined the rs4072037 genotype and measured serum KL-6 levels to evaluate the association between lung adenocarcinoma (ADC) and rs4072037 or serum KL-6 levels. DNA samples were available for 172 patients and these were included in the genomic analyses. In addition, 304 patients were included in the serum analyses. Furthermore, 276 healthy volunteers were included in both genomic and serum analyses.

**Results:**

The rs4072037 genotype was not associated with susceptibility to lung ADC or its prognosis. Interestingly, serum KL-6 levels significantly differed according to rs4072037 genotype in those with T1 or T2 (*P* < 0.001), N0 or N1 (*P* = 0.002) and M0 (*P* < 0.001), but not in those with T3 or T4 (*P* = 0.882), N2 or N3 (*P* = 0.616) and M1a or M1b (*P* = 0.501). Serum KL-6 levels were significantly associated with the presence of lung ADC, as well as with its progression and prognosis, indicating the crucial involvement of KL-6/MUC1 in the development of lung cancer and its progression.

**Conclusion:**

Based on these findings, we conclude that rs4072037 does not have a significant impact on the pathogenesis or prognosis of lung ADC, whereas serum KL-6 levels, which might reflecting the molecular length of MUC1, are significantly associated with lung ADC.

**Electronic supplementary material:**

The online version of this article (doi:10.1186/s12885-017-3272-y) contains supplementary material, which is available to authorized users.

## Background

Lung cancer is one of the most common malignant tumors worldwide. Adenocarcinoma (ADC) is the most common histological type of lung cancer in the United States, Europe, and East Asia, including Japan [1]. Although novel therapeutic targets and molecular-targeted agents have been identified and applied in clinical settings, lung cancer remains the leading cause of cancer death in the majority of developed countries [2]. Thus, the identification of novel diagnostic biomarkers and potential therapeutic target molecules is urgently needed.

Several types of mucins, including mucin 1 (MUC1), are known to contribute to the growth and metastatic properties of various tumors. In addition to their anti-adhesion functions, mucins can affect the transcriptional profile of various gene products involved in cancer cell invasion and metastasis by mediating signal-transduction [3, 4]. These findings indicate the potential utility of MUC1 as a diagnostic biomarker and/or therapeutic target in lung cancer. We have previously demonstrated that *MUC1* variable numbers of tandem repeats (VNTR) polymorphism was significantly associated with susceptibility to lung ADC and its prognosis [5]. However, it would be very difficult to identify *MUC1* gene length polymorphisms in clinical practice. Thus, in the current study, we focused on alternative potential biomarkers which can reflect the VNTR polymorphism and are more easily detectable in the clinical setting: rs4072037, a single nucleotide polymorphism (SNP) in the *MUC1* gene, and serum KL-6 levels.

rs4072037, a functional SNP in exon 2 of the *MUC1* gene, regulates splicing site selection during the post-transcriptional regulation process [6]. It has also been reported that rs4072037 is in linkage disequilibrium with VNTR in *MUC1* [7]. Furthermore, several studies have reported an association between rs4072037 and susceptibility to gastric cancer [8–10]. Although no significant association has been demonstrated between rs4072037 and lung cancer, these previous studies suggest that rs4072037 may play a role in the development of lung ADC and its progression, and that rs4072037 may have utility as a diagnostic and/or prognostic biomarker in lung ADC.

We previously developed a murine IgG1 monoclonal antibody (mAb), designated KL-6, by immunizing a mouse with lung ADC VMRC-LCR cells [11]. The anti-KL-6 mAb recognizes sialylated carbohydrates attached to the core protein of MUC1 through the glycosylation process [12]. KL-6 has been well investigated as a useful diagnostic and prognostic biomarker for various interstitial lung diseases [11, 13, 14]. Additionally, we have demonstrated that KL-6 may be a prognostic biomarker for non-small cell lung cancer patients who have been treated with epidermal growth factor receptor tyrosine kinase inhibitors or surgical resection [15, 16]. While these findings were promising, the number of patients evaluated in these two studies was relatively small. Furthermore, the combined contribution of serum KL-6 with the other important prognostic predictors of lung ADC, such as *EGFR* gene mutation status [17], was not sufficiently evaluated in the previous studies.

The two aims of the present study were as follows: 1) to assess whether rs407247, a SNP in the *MUC1* gene, is associated with lung ADC development or prognosis, and 2) to assess whether KL-6, a carbohydrate associated with MUC1, improves the predictive power of the other predictive factors in patients with lung ADC. In this study, we evaluated the different rs4072037 genotypes and the serum levels of KL-6 in 354 patients with lung ADC and 276 healthy volunteers (HVs).

## Methods

### Patients and clinical samples

**Fig. 1:**
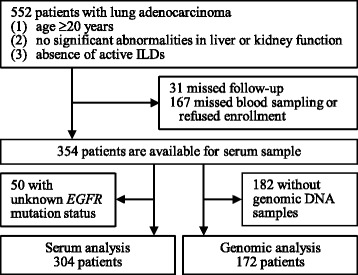
Flow diagram of patient selection. The flow diagram illustrating the inclusion process of the patients with lung adenocarcinoma into the study. Three hundred and four patients were included into serum analysis and 172 patients were included into genomic analysis, with 122 patients included into both analysis

Five hundred and fifty-two patients with previously untreated lung ADC which was pathologically diagnosed and subsequently treated at Hiroshima University Hospital (Hiroshima, Japan) between April 2003 and June 2012 were included in this study (Fig. 1). The inclusion criteria, shown in Fig. 1, were almost identical to those described in our previous studies [15, 16, 18–21]. Disease staging in all 552 cases included computed tomography scans of the chest and abdomen, bone scintigraphy or F-18 fluordeoxyglucose positron emission tomography, and magnetic resonance imaging of the head. The clinical or pathologic TNM stage was determined according to the 7th edition of the TNM classification of malignant tumors [22]. Two hundred and seventy-six HVs who attended health check-ups were also enrolled. The HVs had no history or current diagnosis of malignancy or apparent lung disease. Serum and peripheral venous whole blood samples were obtained from patients and HVs at enrollment with their written informed consents for using their samples and publishing the data. Serum samples were available for 354 of the 552 patients with ADC and for all 276 HVs. Among the 354 patients with ADC, *EGFR* gene mutation status was known for 304 patients that were included in the serum analyses. In addition, DNA samples were available for 172 patients and these were also included in the genomic analyses (Table 1). This study was approved by the Ethics Committee of Hiroshima University Hospital (IRB M33 and 326) and conducted in accordance with the ethical standards established by the Helsinki Declaration of 1975.

**Table 1:** Clinical characteristics

	Patients with lung adenocarcinoma			Healthy volunteers
	All patients	Genomic analysis	Serum analysis	
Subject number	354	172	304	276
Age	65.9 ± 0.6	63.8 ± 0.9	66.6 ± 0.6	49.9 ± 0.4
Gender, Male / Female	195 / 159	95 / 77	164 / 140	228 / 48
Smoking, Yes / No	211 / 143	103 / 69	179 / 125	158 / 118
PS, ≤1 / ≥2	319 / 35	149 / 23	274 / 30	
*EGFR*, Mutant / Wild / ND	122 / 182 / 50	55 / 67 / 50	122 / 182 / 0	
T factor, ≤2 / ≥3	237 / 117	105 / 67	207 / 97	
N factor, ≤1 / ≥2	180 / 174	72 / 100	156 / 148	
M factor, 0 / 1	189 / 165	78 / 94	160 / 144	

*PS* performance status, *ND* not detected

### *EGFR* mutation status

*EGFR* mutation status was evaluated in 304 patients, for whom serum samples were available, in one of the following sample types: cytology liquid samples derived from bronchoscopy or thoracentesis, paraffin-embedded transbronchial lung biopsy samples, or surgically resected tumor tissues. To evaluate *EGFR* mutations, the peptide nucleic acid-locked nucleic acid polymerase chain reaction clamp test that detects the G719C, G719S, G719A, L858R, L861Q, and T790 M mutations and seven different exon 19 deletions was used, as previously described [23].

### DNA preparation and rs4072037 genotyping

DNA was extracted from peripheral venous whole blood samples using the phenol-chloroform extraction and ethanol precipitation methods, as previously described [24–26]. The rs4072037 genotype was determined by real-time polymerase chain reaction (PCR) using a commercially available SNP genotyping assay (TaqMan SNP Genotyping Assay C 27532642–10; Life Technologies Corp. Carlsbad, CA, USA) and the Applied Biosystems 7500 Fast RT-PCR System (Life Technologies Corp.)

### Measurement of serum KL-6 levels

Serum KL-6 levels were measured with a sandwich-type electrochemiluminescence immunoassay on a Picolumi 8220 Analyzer (Sanko Junyaku, Tokyo, Japan), as previously described [15, 16, 20, 27].

### Statistical analysis

The numerical data were presented as the mean ± standard error of the mean. The Mann–Whitney *U*-test was used to analyze data between 2 groups; for 3 or more groups, the Kruskal-Wallis test followed by multiple comparisons using rank sums was performed [28]. When dividing patients into clinical subgroups according to performance status (PS) or TNM factors, we performed receiver operating characteristic (ROC) analysis and determined the appropriate cut-off point to predict the prognosis of the patients. Survival analysis was performed using the log-rank test and Cox proportional hazards models. Concordance (C)-statistics were used to evaluate and compare the Cox models. To test for deviations from Hardy-Weinberg equilibrium, genotype frequencies were determined by direct counting and the chi-square test or Fisher’s exact test were used as appropriate. Allelic frequencies were calculated based on the genotype frequencies, and the association between the minor G allele of rs4072037 and lung ADC was tested using the chi-square test or Fisher’s exact test. All statistical analyses were performed using SPSS for Windows, version 18.0 (SPSS Inc. Chicago, USA).

## Results

### The rs4072037 genotype was not associated with susceptibility to lung ADC or its prognosis

To investigate the association between rs4072037 and susceptibility to lung ADC and/or its prognosis, we extracted DNA from peripheral venous blood samples and evaluated the presence of the rs4072037 genotype in 172 ADC patients and 276 HVs. The clinical characteristics of the studied subjects are shown in Table 1. The HVs were predominantly male and relatively young, compared with patients with lung ADC. The characteristics of patients with ADC included in the genomic analyses and those included in the serum analyses were similar. As shown in Table 2, the genotype distributions of rs4072037 were in Hardy–Weinberg equilibrium for both patients and HVs (*P* = 0.891 and *P* = 0.979, respectively). Unexpectedly, the genotype distributions of rs4072037 did not differ significantly between patients with ADC and HVs (*P* = 0.933). This result was consistent even when we limited the patients to those with advanced stage disease (Table 2). In addition, genotypes of rs4072037 showed no significant association with the risk of lung ADC both in dominant and recessive models (Table 3). According to the Kaplan-Meier analysis, the mean survival times for patients with AA, AG, and GG genotypes were 2416.8 ± 257.2 days, 1988.8 ± 232.5 days and 486.0 ± 206.8 days, respectively. Patients with the GG genotype tended to have a poorer prognosis than the other patients, although this was not statistically significant according to the log-rank test (*P* = 0.173). We also have to pay attention to the fact that there was only 4 patients with the GG genotype.

**Table 2:** Genotype distributions of rs4072037

	AA (%)	AG (%)	GG (%)	HWE	chi-square
Healthy volunteers	186 (67.4)	82 (29.7)	8 (2.9)	0.979	ref.
All patients	116 (67.5)	52 (30.2)	4 (2.3)	0.891	0.933
limited to ≥T3	46 (68.7)	21 (31.3)	0 (0)	0.376	0.367
limited to ≥N2	65 (65.0)	33 (33.0)	2 (2.0)	0.772	0.761
limited to ≥M1a	62 (66.0)	28 (29.8)	4 (4.2)	0.968	0.811

*HWE* Hardy-Weinberg equilibrium

**Table 3:** rs4072037 genotype and the risk of lung adenocarcinoma

	Dominant model		Recessive model	
	OR (95% CI)	*P* value	OR (95% CI)	*P* value
All patients	0.998 (0.665–1.498)	0.991	0.798 (0.237–2.690)	0.484
limited to ≥T3	0.943 (0.531–1.675)	0.843	0.971 (0.951–0.991)	0.172
limited to ≥N2	1.113 (0.687–1.802)	0.664	0.684 (0.143–3.275)	0.477
limited to ≥M1a	1.067 (0.650–1.750)	0.798	1.489 (0.438–5.062)	0.363

*OR* odds ratio, *CI* confidence interval

### Serum KL-6 levels varied according to the rs4072037 genotype in HVs and patients with early stage ADC, but not in those with advanced disease

**Fig. 2:**
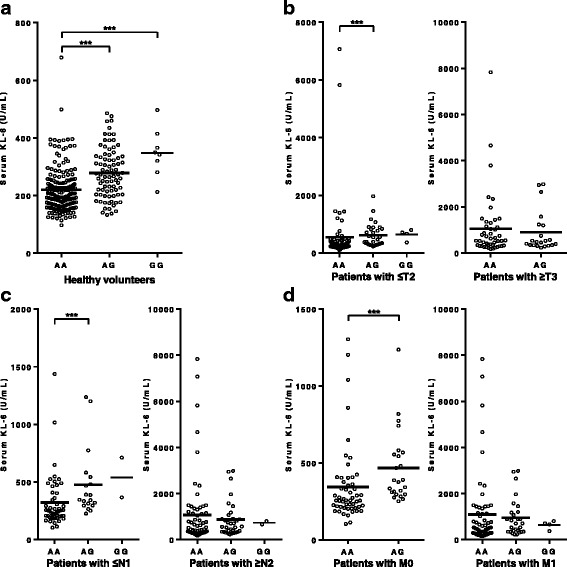
Serum KL-6 according to the genotypes of rs4072037. The distributions of serum KL-6 levels according to the genotypes of rs4072037 **a** in healthy volunteers, **b** in the patients with T1 or T2 (*left panel*) and with T3 or T4 (*right panel*), **c** in the patients with N0 or N1 (*left panel*) and with N2 or N3 (*right panel*), and **d** in the patients with M0 (*left panel*) and with M1a or M1b (*right panel*). The *horizontal bars* represent the mean values. ***P < 0.001 (Mann-Whitney *U* test)

Next, we investigated the correlation between the rs4072037 genotype and serum KL-6 levels. As shown in Fig. 2a, there was a significant correlation between the rs4072037 genotype and serum KL-6 levels in HVs, which was in line with the results of previous studies [24, 29]. In patients with lung ADC, a significant correlation between the rs4072037 genotype and serum KL-6 levels was observed in those with T1 or T2 (Fig. 2b, *P* < 0.001), N0 or N1 (Fig. 2c, *P* = 0.002) and M0 (Fig. 2d, *P* < 0.001), but not in those with T3 or T4 (Fig. 2b, *P* = 0.882), N2 or N3 (Fig. 2c, *P* = 0.616) and M1a or M1b (Fig. 2d, *P* = 0.501).

### Serum KL-6 levels were significantly elevated in patients with lung ADC

**Fig. 3:**
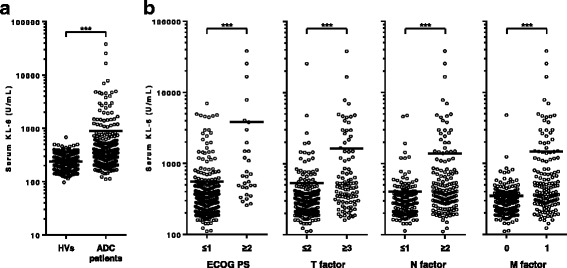
Serum KL-6 according to the presence or the clinical stage of lung adenocarcinoma. The distributions of serum KL-6 levels according to **a** the presence or absence of lung adenocarcinoma, **b** performance status and TNM classifications are presented as scattered plots. HVs, healthy volunteers; ADC, adenocarcinoma; PS, performance status. The *horizontal bars* represent the mean values. ***P < 0.001 (Mann-Whitney *U* test)

To confirm the correlation between serum KL-6 levels and the presence of lung ADC, we compared serum KL-6 levels between 304 patients with ADC and 276 HVs. As shown in Fig. 3a, serum KL-6 levels were significantly higher in patients with ADC than in HVs (880.8 ± 166.3 U/mL and 241.0 ± 5.2 U/mL, *P* < 0.001). In addition, high serum KL-6 levels were associated with poor PS (*P* < 0.001, Fig. 3b), and advanced T, N and M factors (*P* < 0.001, *P* < 0.001 and *P* < 0.001, respectively; Fig. 3b). On the other hand, serum KL-6 levels did not differ between patients with ADC with wild-type *EGFR* and mutant *EGFR* status (768.4 ± 170.4 U/mL and 1048.5 ± 327.7 U/mL, *P* = 0.920, Additional file 1).

### Serum KL-6 levels significantly correlated with survival in patients with lung ADC

**Fig. 4:**
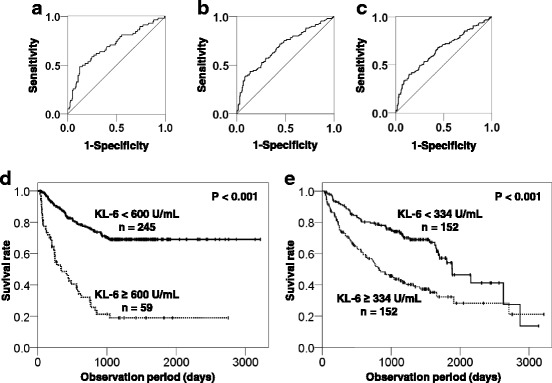
Serum KL-6 can predict the outcome of patients with lung adenocarcinoma. Receiver operating characteristic curves for (**a**) 1-year, (**b**) 3-year, and (**c**) 5-year survival. The Kaplan-Meier survival curves for each cohort stratified by the two different cut-off value of serum KL-6; (**d**) 600 U/mL (set by ROC analyses), (**e**) 334 U/mL (median value)

We performed ROC analysis to evaluate the predictive ability of serum KL-6 in patients with ADC. The area under the curve for 1-year, 3-year, and 5-year survival was 0.698 (95% confidence interval [CI] = 0.618–0.778; *P* < 0.001, Fig. 4a), 0.686 (95% CI = 0.622–0.749; *P* < 0.001, Fig. 4b) and 0.656 (95% CI = 0.593–0.719; *P* < 0.001, Fig. 4c), respectively. The optimal cut-off value for serum KL-6 levels to discriminate between survivors and non-survivors was set at 600 U/mL, in accordance with the ROC analyses. According to the Kaplan-Meier analysis, the mean survival times for patients with higher and lower serum KL-6 levels were 777.8 ± 117.7 days and 2807.6 ± 224.2 days, respectively. Patients with higher serum KL-6 levels had a significantly poorer prognosis than those with lower serum KL-6 levels, as tested by the log-rank test (*P* < 0.001, Fig. 4d). This result was confirmed when we perform additional log-rank test stratified by median value of serum KL-6 (*P* < 0.001, Fig. 4e). To further investigate the predictive ability of serum KL-6, we performed Cox proportional hazards regression analysis with stepwise selection including both patients with mutant *EGFR* and those with wild type *EGFR*. The initial covariates included age, gender, smoking history, PS, *EGFR* mutation status, TNM classifications and serum KL-6 levels. As shown in Table 4A, there was a significant association between serum KL-6 levels and prognosis in patients with ADC, independent of the other covariates (PS, *EGFR* mutation status, and N and M factors). In addition, this result was not changed when we forcibly include age or gender into the covariates (data not shown). Furthermore, comparison of the C-statistics of each prediction model, with or without serum KL-6, demonstrated a significant increase in C-index with the addition of serum KL-6 to the other covariates (Table 4B).

**Table 4:** Impact of serum KL-6 on the prognosis of lung adenocarcinoma

Variable	HR	95% CI.	*P* value
A. Multivariate Cox proportional hazards regression model			
PS, ordinal	1.72	1.35–2.20	<0.001
*EGFR*, wild type / mutant	2.43	1.62–3.64	<0.001
N factor, ordinal	1.23	1.04–1.45	0.018
M factor, yes / no	3.33	2.02–5.48	<0.001
Serum KL-6, ≥600 / <600 U/mL	2.19	1.43–3.37	<0.001
B. Comparison of C statistics for each prediction model			
Prediction model	C index	95% CI.	*P* value
Covariates only	0.776	0.722–0.830	Ref.
Covariates + serum KL-6	0.790	0.736–0.843	0.036

“Covariates” in C statistics included PS, *EGFR* mutation status, and N and M factors. *PS* performance status

## Discussion

In the present study, no significant association was found between the rs4072037 genotype and susceptibility to lung ADC or its prognosis. rs4072037 genotype significantly affected serum KL-6 levels in patients with early stage ADC, but not in those with advanced disease. Furthermore, we demonstrated that serum KL-6 levels were significantly associated with the presence of lung ADC and its prognosis.

Although the *MUC1* VNTR polymorphism is associated with susceptibility to lung ADC and its prognosis [5], we found that rs4072037, a SNP in the *MUC1* gene, was not associated with susceptibility to lung ADC or with its prognosis. MUC1 is known to inhibit E-cadherin-mediated cell-cell adhesion and integrin-mediated cell-extracellular matrix adhesion [30, 31]. It is also known that MUC1 is involved in the growth and metastasis of various tumors, mainly due to its anti-adhesion and signal-transduction functions [3, 4]. We have previously reported that lung ADC cells expressed high levels of KL-6/MUC1 [16, 32] and that an anti-KL-6 mAb induced capping of MUC1, thereby interfering with its anti-adhesion function and inhibiting tumor proliferation [33]. On the basis of these previous findings, we hypothesized that the rs4072037 genotype in MUC1 was associated with susceptibility to lung ADC and/or its prognosis. However, we failed to verify our hypothesis. The link between rs4072037 and the VNTR polymorphism, which was reported in 1996 [7], has actually been challenged by several recent studies. Imbert et al. reported that the MUC1 VNTR polymorphism correlated with the rs4072037 genotype in only 80% of dry eye cases [34]. A similar result in patients with sarcoidosis was also reported by Shigemura et al. [35]. Based on these findings, we speculate that the VNTR polymorphism in MUC1, but not the rs4072037 genotype, has a significant impact on the pathogenesis and progression of lung ADC.

Interestingly, we found that the correlation between the rs4072037 genotype and serum KL-6 levels, which was previously reported [24, 29, 35], was significant in HVs and in patients with early stage ADC, but not in those with advanced ADC (Fig. 2). Serum KL-6 in patients with lung ADC, especially in those with advanced disease, is considered to be predominantly derived from lung cancer cells with a minor contribution from normal type II pneumocytes, which are known to be major source of serum KL-6 under normal conditions [16, 32]. In addition, serum KL-6 levels have been reported to be influenced by the molecular size of MUC1 in healthy controls or in patients with sarcoidosis [35]. However, such a correlation has not been reported in patients with lung cancer. Therefore, we can speculate that the rs4072037 genotype has a significant impact on serum KL-6 derived from normal type II pneumocytes, but not on serum KL-6 derived from lung ADC cells. In cancer cells, alterations in the glycosylation properties of mucins, which encompass aberrant glycosylation of *O*-linked glycans, are known to play an important role in tumorigenesis, tumor progression, and metastasis [36–41]. Serum KL-6 levels in patients with lung ADC may be influenced by the aberrant glycosylation of *O*-linked glycans in MUC1 and also by the depolarized expression pattern of MUC1 in lung ADC [12, 16]. The influence of these epigenetic factors may diminish the association between the rs4072037 genotype and serum KL-6 levels in patients with advanced ADC.

In the present study, we successfully confirmed that serum KL-6 levels are independently associated with prognosis in patients with lung ADC. Although the correlation between serum KL-6 levels and disease progression or prognosis in patients with lung ADC has already been reported [11, 15, 16], the results from the present study are still of pivotal importance; we have demonstrated that serum KL-6 is a significant prognostic factor for lung ADC, independent of the other known prognostic factors, including *EGFR* mutation status [17]. Based on these data, it is clear that KL-6/MUC1 plays an important role in tumor cell growth, proliferation, and metastasis. Therefore serum KL-6 may be an alternative and useful biomarker; it can reflect the VNTR polymorphism in MUC1 and is more easily detectable in the clinical setting.

This study had some limitations. First, the patient sample size was small, especially for the genomic analysis. Considering the low frequency of the rs4072037 G allele, we cannot exclude the possibility that the small number of patients harboring the G allele influenced the results. Second, age and sex distributions differed between patients and HVs. Despite these limitations, the results from our study are quite evocative and provide important insight into the crucial involvement of KL-6/MUC1 in the development of lung cancer and its progression.

## Conclusions

In conclusion, we demonstrated that rs4072037, a SNP in *MUC1* gene, does not affect lung ADC development or its prognosis. We also demonstrated that serum KL-6 levels were significantly associated with the presence of lung ADC and its progression and prognosis, indicating the crucial role of KL-6/MUC1 in the development of lung cancer and its progression.

## Additional files

Additional file 1: Serum KL-6 according to the presence or absence of *EGFR* gene mutation. (PDF 20 kb)

Additional file 2: The dataset supporting the conclusions of this article is available in this datasheet. (XLS 133 kb)

## Abbreviations

ADC: Adenocarcinoma
CI: Confidence interval
HV: Healthy volunteer
mAb: monoclonal antibody
MUC1: Mucin 1
PCR: Polymerase chain reaction
PS: Performance status
ROC: Receiver operating characteristic
SNP: Single nucleotide polymorphism
VNTR: Variable numbers of tandem repeats
