# Supplementary figures and images for: MUC1 in lung adenocarcinoma: cross-sectional genetic and serological study
Source: BMC Cancer. 2017 Apr 12;17:263. doi: 10.1186/s12885-017-3272-y (PMC5388999; doi:10.1186/s12885-017-3272-y)

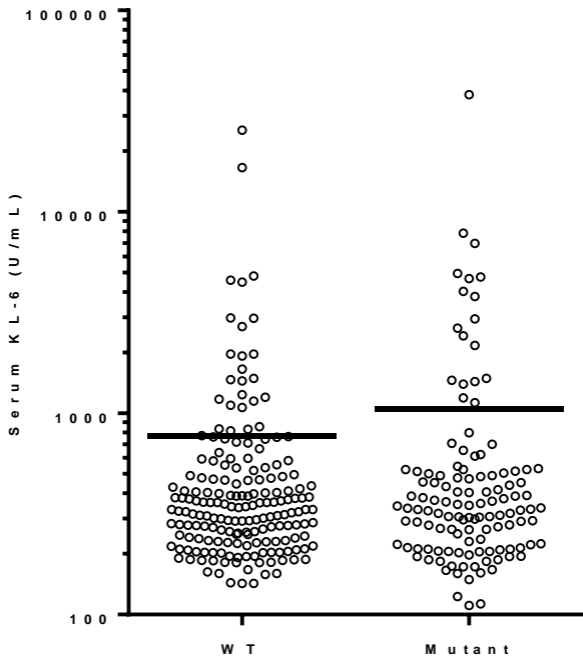

Supplement: Supplementary file 1 — Serum KL-6 according to the presence or absence of EGFR gene mutation. (PDF 20 kb) [file 12885_2017_3272_MOESM1_ESM.pdf]
